# When Everything Revolves Around Internal Carotid Artery: Analysis of Different Management Strategies in Patients With Very Advanced Cancer Involving the Skull Base

**DOI:** 10.3389/fonc.2021.781205

**Published:** 2021-11-18

**Authors:** Ester Orlandi, Marco Ferrari, Elvis Lafe, Lorenzo Preda, Marco Benazzo, Barbara Vischioni, Maria Bonora, Vittorio Rampinelli, Alberto Schreiber, Lisa Licitra, Piero Nicolai

**Affiliations:** ^1^ Radiation Oncology Clinical Department, National Center for Oncological Hadrontherapy (“Fondazione CNAO”), Pavia, Italy; ^2^ Section of Otorhinolaryngology – Head and Neck Surgery, Department of Neurosciences, University of Padua – “Azienda Ospedaliera di Padova”, Padua, Italy; ^3^ University Health Network (UHN) Guided Therapeutics (GTx) Program International Scholar, UHN, Toronto, ON, Canada; ^4^ Technology for Health (PhD Program), Department of Information Engineering, University of Brescia, Brescia, Italy; ^5^ Department of Diagnostic Radiology and Interventional Radiology and Neuroradiology, University of Pavia, IRCCS Policlinico San Matteo Foundation, Pavia, Italy; ^6^ Department of Clinical-Surgical, Diagnostic and Pediatric Sciences, University of Pavia, IRCCS Policlinico San Matteo Foundation, Pavia, Italy; ^7^ Department of Otorhinolaryngology, University of Pavia, IRCCS Policlinico San Matteo Foundation, Pavia, Italy; ^8^ Unit of Otorhinolaryngology – Head and Neck Surgery, University of Brescia – “ASST Spedali Civili di Brescia”, Brescia, Italy; ^9^ Department of Oncology and Hematology-Oncology, University of Milan, IRCCS Istituto Nazionale dei Tumori, Milan, Italy

**Keywords:** head & neck, cancer, encasement, involvement, carotid, skull base (head and neck)

## Abstract

Internal or common carotid artery encasement (CAE) is observed in almost 2-7% of head and neck cancers (HNC) and designates the tumor with the T4b category. This clinical scenario is associated with a dismal prognosis, owing to the risk for thrombosis and bleeding that usually characterizes such an advanced cancer. Standardized radiological criteria to infer invasion of the carotid artery are lacking. Complete surgical resection in the context of a multimodality treatment is supposed to offer the greatest chances of cure. Surgery can either be carotid-sparing or include carotidectomy. Data on probability of cerebrovascular and non-cerebrovascular complications, risk of carotid blowout, poor oncologic outcomes, and less-than-certain efficacy of diagnostic and interventional preventive procedures against cerebral infarction make it difficult to define surgery as the recommended option among other therapeutic strategies. Non-surgical therapies based on radiation therapy possibly combined with chemotherapy are more frequently employed in HNC with CAE. In this context, carotid blowout is the most feared complication, and its probability increases with tumor stage and cumulative radiation dose received by the vessel. The use of highly conformal radiotherapies such as intensity-modulated particle therapy might substantially improve the manageability of HNC with CAE by possibly reducing the risk of late sequalae. Despite evidence is frail, it appears logical that a case-by-case evaluation through multidisciplinary decision making between head and neck surgeons, radiation oncologists, medical oncologists, diagnostic and interventional radiologists, and vascular surgeons are of paramount value to offer the best therapeutic solution to patients affected by HNC with CAE.

## Introduction

Internal or common carotid artery encasement (CAE) by head and neck cancers (HNCs), including salivary gland and sinonasal cancers, designates the tumor with the T4b category (1). Carotid artery (CA) can be encircled, and its walls potentially invaded by the primary tumor and/or nodal metastases with extranodal extension. CAE has a low but non-negligible incidence, accounting for approximately 2–7% of advanced HNCs (2), more often in patients affected by recurrent or persistent disease. In all cases, a comprehensive, multidisciplinary plan is necessary to pursue the optimal patient-centered approach (3). This clinical scenario is generally associated with a very poor prognosis, which is determined by both the risk for fatal exsanguination when cancer erodes CA walls and the abrupt tumor progression which usually characterizes such an advanced neoplastic stage (4, 5). Moreover, CAE is usually not specified as an inclusion or stratifying criteria in trials assessing the role of non-surgical therapies.

Criteria to define CAE at imaging, mastering various therapeutic approaches for locally advanced disease with CAE, management options to prevent CA blowout (CB), as well as knowledge of potential cerebrovascular risks related to permanent CA occlusion are all contemporary aspects that should raise the interest of different physicians who deal with advanced HNCs. The present perspective will emphasize on these aspects based on the most relevant current evidence.

## CAE Definition Criteria

So far, there are no standardized radiological criteria to distinguish simple CAE from frank vessel involvement. Preoperative magnetic resonance imaging (MRI) has been demonstrated capable in predicting involvement of the CA in cases with near-circumferential encasement (6). In contrast, despite computed tomography (CT) is more commonly used to stage HNCs, its utility in predicting CA invasion has been questioned (7). In 1995, Yousem et al. analyzed 49 MRI (53 CAs) and reported that when tumor surrounded the CA for <180° or between 180–270°, no CA invasion was found at surgery. When focusing on cases with >270° encasement, CA invasion was observed in 71% of patients (6). In 2010, Pons et al. reported on 22 patients preoperatively staged through both CT and MRI. They found that combination of CA deformation, >180° encasement, and segmental obliteration of the fat separating the vessel from the adenopathy or primary tumor was highly predictive of invasion of the CA wall. On the other hand, the isolated finding of >180° encasement or fat obliteration could not reliably indicate an invasion of the CA (8). Other studies reported similar results (2, 9, 10). However, standardized and validated radiological criteria to infer CA invasion are lacking. Thus, radiological definition of CAE is challenging and not founded upon sound data. Therefore, beside counting on a head and neck imaging-trained radiologist, the multidisciplinary team should also include vascular surgeons and interventional radiologists and be equipped with the necessary resources to assess on a case-by-case basis the resectability and curability of a CA-encasing tumor.

## Therapeutic Options for Patients With CA-Abutting Cancers and Their Complications

**Figure 1:**
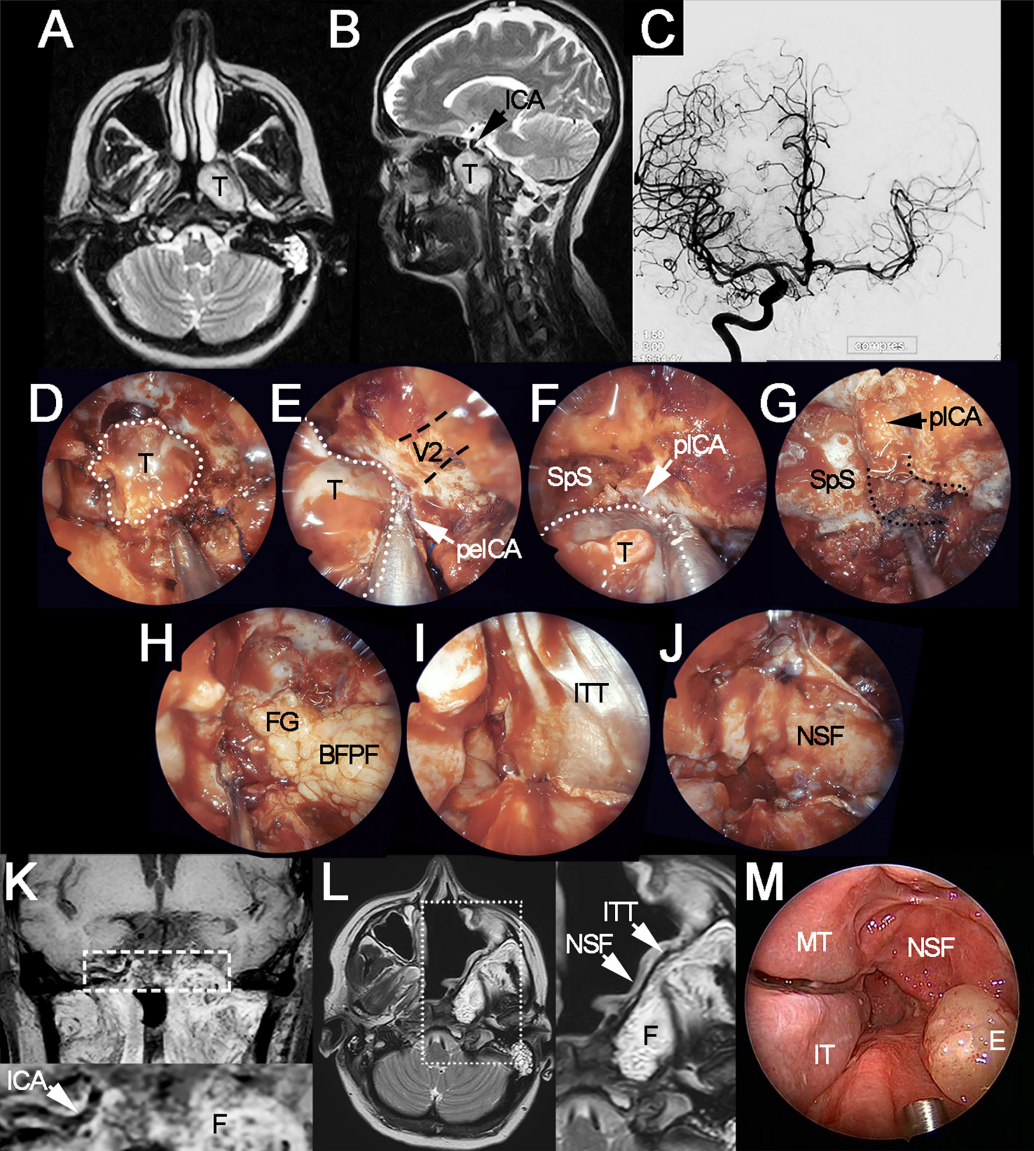
Locally advanced polymorphous adenocarcinoma of the left nasopharyngeal wall-sphenoid sinus, treated through left extracranial-to-intracranial bypass surgery, endoscopic transnasal resection, and adjuvant intensity-modulated radiation therapy. **(A, B)** Preoperative axial and sagittal T2-weighted MRI showing the tumor (T) and its spatial relationship with the internal carotid artery (ICA). **(C)** Angiography of the temporary balloon occlusion test, which showed adequate crossflow timing but was considered as positive for ischemia due to neurological signs at sensitization through drug-induced hypotension. **(D)** Endoscopic appearance of the tumor (white dotted line) after sphenoidotomy, ethmoidectomy, and medial maxillectomy. **(E, F)** Intraoperative evaluation confirmed the presence of tight adhesion to the caudal paraclival (pICA) and medial petrous (peICA) tracts of the internal carotid artery. **(G)** Endoscopic view of the surgical field following carotidectomy, occlusion coils can be sees as emerging from the distal stump of the vessel (i.e., cranial paraclival tract). Clearance of the carotid canal (black dotted lines) and petroclival junction have been performed. **(H–J)** Reconstruction of the skull base defect through the right buccal fat pad flap (BFPF), fat graft (FG), iliotibial tract graft (ITT), and the right nasoseptal flap (NSF). **(K)** Coronal T1-weighted, fat-saturated, contrast-enhanced MRI acquired 10 months after surgery (7 months after completion of adjuvant radiation therapy). White dashed rectangle indicates the position of magnification at the bottom of the image, which shows the right internal carotid artery and enhancing fat **(F)** in the position of the left carotid canal and petroclival junction. **(L)** T2-weighted MRI acquired 10 months after surgery. White dotted rectangle indicates the position of magnification on the right of the image, which shows the layers of the reconstruction. **(M)** Endoscopic appearance of the surgical site 10 months after surgery. V2, position of the maxillary nerve (black dashed lines indicate the trajectory of the nerve); E, mucosal edema; IT, inferior turbinate; MT, middle turbinate; SpS, sphenoid sinus.

The therapy reported to offer the greatest chances of cure in patients with resectable HNC with CAE is surgery aimed to obtain a complete tumor resection, which can be achieved through sub-adventitial dissection or CA resection when the tumor only abuts or frankly invade/encase the vessel, respectively (**Figure 1**). The most relevant, life-threatening complication of CA-sparing surgery is CB, which has an average incidence of 3-4.5% (0-2.4% in naïve patients, 4.5-21.1% in previously irradiated patients) and mean lethality rate as high as 50% (11). In an animal study published in the 1970s, sub-adventitial dissection to peel the tumor off CA combined with infection of the surgical site have been hypothesized as being the main determinants of postoperative CB (12). Thus, one could hypothesize abutted CA to be resected irrespective of its genuine invasion by cancer, with the twofold advantage of preventing CB and providing a wider margin of resection. However, resecting the CA does not compensate the advanced stage and biological aggressiveness of HNC determining CAE. In fact, several series of HNCs, mostly represented by squamous cell carcinoma (SCC), treated through CA resection-including surgery reported a 2-year overall survival rate as low as 11.1-50.0% (13–16). On the other hand, perioperative mortality (10-25%) (17–19) and risk for cerebrovascular (12.5-33%) (13–16) and non-cerebrovascular complications (25-60%) (13, 14, 16, 20) are non-negligible in patients receiving CA resection-including surgery. Despite cerebral revascularization is supposed to reduce the incidence of cerebrovascular events (19, 21), the comparative study by Aslan et al. was unable to demonstrate a significant difference (17). Cerebral revascularization can be achieved through either CA reconstruction, which is technically feasible when common CA and/or extracranial (i.e., parapharyngeal) internal CA are resected, or bypass surgery, which consists of creating a communication between a donor arterial system, such as the external carotid one, and the cerebral vascularization (e.g., to the middle cerebral artery) *via* an interpositional vascular graft. Moreover, prior to indicate CA resection without cerebral revascularization in a patient tolerating a temporary balloon occlusion test, one should consider that the rate of delayed cerebrovascular events in patients with negative occlusion test accounts for 15-22% (22–24). Of note, more than one study demonstrated that morbidity and mortality in patients receiving CA resection-including surgery had a decreasing trend after the 1990s (18, 19). These data taken altogether suggest that only meticulous selection of patients and minimization of surgical morbidity, which cannot prescind from involvement of a neurologist and neurosurgeon in the multidisciplinary team, could lead CA resection-including surgery to be a valuable therapeutic option for some patients with CAE-determining HNC. As an example of the need to accurately select patients, Yokoyama et al. reported a series of 10 patients receiving CA resection and reconstruction through a superficial femoral vein graft: the 5-year overall survival rates of patients affected by SCC and non-SCC cancers were <20% *versus* 100%, respectively (25). These data witness that histology represents one of the factors to consider when CA resection-including surgery is proposed.

Nonsurgical modalities, mainly represented by photon-based radiotherapy (RT), delivered either alone or in combination with chemotherapy (CRT), are aimed at avoiding complications of CA resection. An HNC may be labelled as “unresectable” either because genuinely unsuitable for a wide-margin resection (i.e., invasion of the skull base, nasopharynx, prevertebral space or cervical spine, fixation of nodes, massive bilateral nodal involvement) or due to the estimated unfavorable balance between risks of surgery and its potential benefits from an oncologic standpoint. Over one-third of these patients are usually treated by neoadjuvant chemotherapy followed by CRT (26–29). However, there are few data regarding the outcome of nonsurgical treatments in patients affected by HNC with CAE. Roh et al. reported a cohort of patients with CA-invading HNC: the median survival was 16.5 months in patients treated with surgery (n=11) with or without reconstruction or ligation of the CA, possibly combined with (neo)adjuvant (C)RT, 11.5 months for patients receiving definitive (C)RT (n=6), and 3 months for those treated palliatively (n=6) (p<0.05). CA was not occluded in patients receiving RT, some of them undergoing a temporary balloon occlusion test prior to treatment (5). In addition, no separate outcome analysis was performed for naïve and recurrent patients. Manzoor et al. also reviewed the outcomes of 44 consecutive *de novo* and recurrent HNCs patients with CA involvement. Survival outcome was not significantly different between patients treated with definitive CRT and surgery with or without postoperative RT (p=0.47), although a trend was found in favor of CRT, possibly because of the treatment-naïve nature of these patients. Of note, imaging was assessed in 7/8 patients treated with radical CRT, and all had near-total circumferential CAE (30).

**Figure 2:**
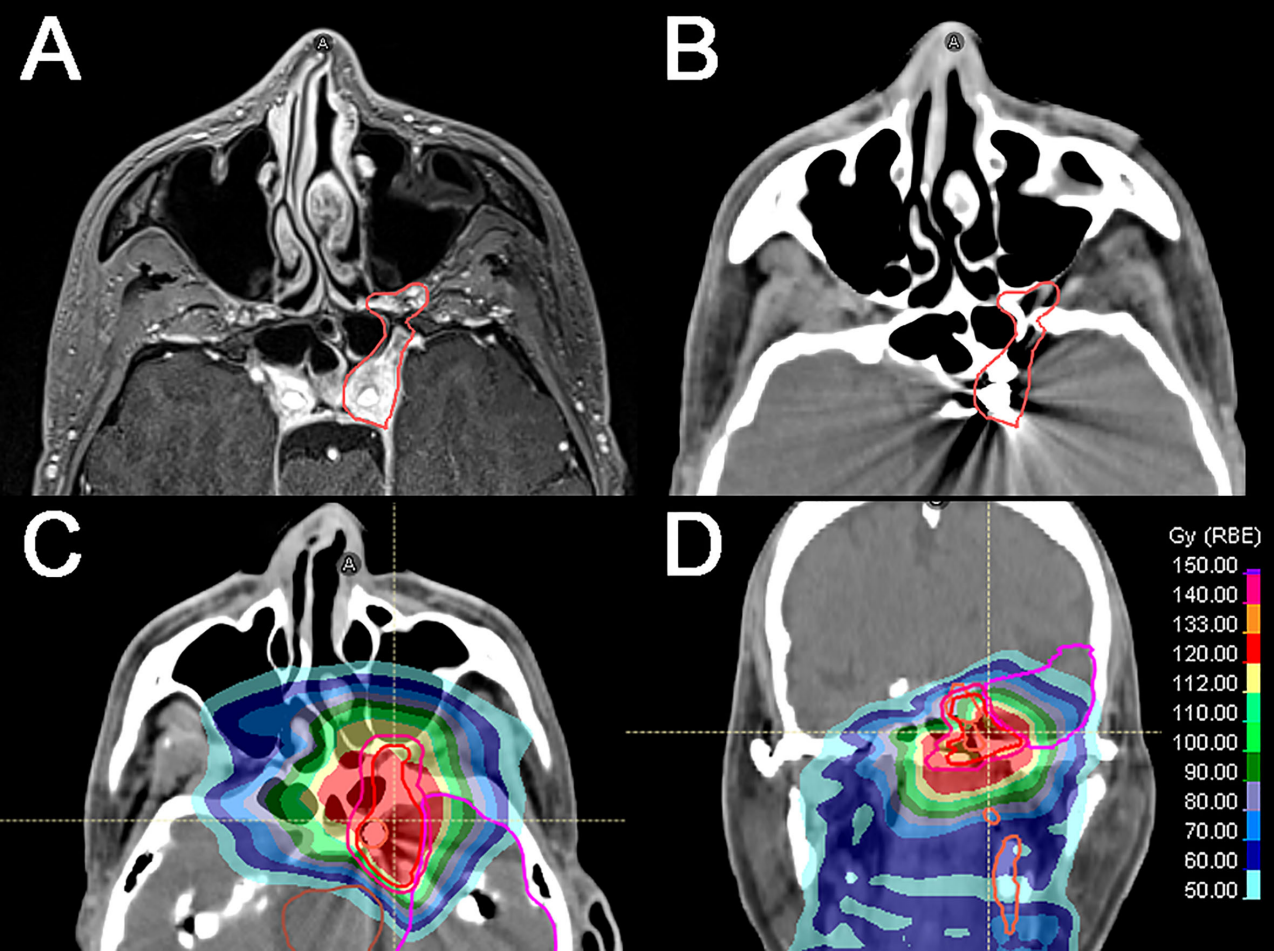
Locally recurrent nasopharyngeal squamous cell carcinoma diagnosed 5 years after the initial diagnosis (cT3N1). **(A)** Axial T1-weighted, fat-saturated, contrast-enhanced image shows the recurrence (*red line*) extending through the left foramen rotundum and vidian canal and involving the foramen ovale and cavernous sinus. Left cavernous internal carotid artery (ICA) is encased by the tumor. **(B)** Axial CT simulation image showing the tumor (red line) and the left ICA occluded with endovascular coils following a well-tolerated temporary balloon occlusion test. **(C, D)** CT axial and coronal images showing the tumor and the cumulative dose distribution according to the primary photon RT plan and the definitive re-irradiation through intensity-modulated proton therapy (54 GyE). Isodose levels are represented by different colors.

No data on CB events were reported in non-surgically treated patients in these two latter series (5, 30). However, CRT can determine the obliteration of the carotid vasa vasorum, leading to fibrosis of the adventitia and subsequent weakening of the arterial wall (31). Indeed, in a study on 1072 patients receiving CRT with conventional fractionation for HNC, the cumulative incidence of CB increased stepwise from 1.4% to 6.1% considering T1 to T4 cancers, suggesting that locally advanced tumors are associated with a higher risk of CB (32). The overall incidence of CB further increases in patients undergoing re-irradiation for HNC (11, 33), with CAs receiving a cumulative dose of 120 Gy or higher blowing out in 25% of cases within 1 year (34). Of note, the highest CB rates published were in patients affected by recurrent nasopharyngeal cancer re-irradiated through hypofractionated stereotactic RT. These data pose a considerable dilemma to the radiation oncologist, who is forced to either delivering a suboptimal dose or putting the patient at risk of CB, particularly in the re-irradiation setting. This concern could be tempered when delivering high precision RT, like protons and carbon ions. High-linear energy transfer carbon ions RT (CIRT) has recently entered the clinical practice. It enables dose escalation due to specific ions physical properties (allowing highly conformal dose distributions) and offers superior relative biological effectiveness by at least a 2-3-fold factor in comparison to conventional RT (35). There is some evidence that radioresistant HNCs and skull base tumors, such as adenoid cystic carcinoma, may benefit from CIRT, usually using hypofractionated regimen, in terms of outcome and safety. This is particularly true in inoperable/unresectable tumors, macroscopic residues, and recurrences (36–38). So far, there are scant data on the occurrence of vascular complications after CIRT. Jensen et al. reported a CB incidence of 3.8% in 52 patients receiving CIRT-based re-irradiation for recurrent adenoid cystic carcinoma. One-year local control and overall survival were 70.3% (2-year estimate: 47.4%) and 81.8% (2-year estimate: 63.3%), respectively, which is higher compared to conventional RT (39). In another paper, only 1/229 patients re-treated with CIRT had a CB. This was a patient with a recurrent adenoid cystic carcinoma of the right base of the skull already treated with 2 courses of RT. The patient recovered quickly from a post-interventional stroke and survived for 9 months after CB. Median local progression-free survival after CIRT was 24.2 months and the median overall survival 26.1 months (40). Neither of these two studies analyzed the radiological relationship between CA and the tumor, nor was the possibility to stent or occlude CA before starting re-treatment discussed. The latter strategy should be considered in view of the potential benefits in terms of local control and survival in certain histologies candidates to CIRT (**Figure 2**).

## Conclusions

Patients with naïve or recurrent advanced HNC with CAE may still have a chance to be cured if treated with modern surgical and RT techniques. In particular, this should be taken into account in young patients with a relatively indolent disease, who may potentially have a relatively long life expectancy. In this clinical scenario, a careful evaluation of the available management strategies to secure CA should be put in place to achieve the best oncological results while minimizing the risk of cerebrovascular and non-cerebrovascular sequelae. Precise analysis of tumor extension, adequate treatment planning, and proper counseling should save patients an ineffective invasive treatment, such as an unintentional R2 surgery, and the risk of dangerous and potentially life-threatening complications, such as CB. These prerequisites are best fulfilled in tertiary referral centers, where the multidisciplinary team can handle very advanced cancers with CAE by exploiting the available strategies and customizing treatment based on special characteristics of the single case. It is authors’ opinion that prospective studies are needed to objectively assess the risk-benefit ratio of CA securing strategies (e.g., stenting or occlusion) that are adopted to deliver the locoregional treatment of HNC with CAE.

## Data Availability Statement

The original contributions presented in the study are included in the article/supplementary material. Further inquiries can be directed to the corresponding author.

## Author Contributions

Authors contributed as follows: conception (EO, MF, EL, LP, MBe, BV, MBo, VR, AS, LL, and PN). Perspective design (EO and MF). Paper draft (EO and MF). Draft correction (EL, LP, MBe, BV, MBo, VR, and AS). Supervision (LL and PN). All authors contributed to the article and approved the submitted version.

## Conflict of Interest

The authors declare that the research was conducted in the absence of any commercial or financial relationships that could be construed as a potential conflict of interest.

## Publisher’s Note

All claims expressed in this article are solely those of the authors and do not necessarily represent those of their affiliated organizations, or those of the publisher, the editors and the reviewers. Any product that may be evaluated in this article, or claim that may be made by its manufacturer, is not guaranteed or endorsed by the publisher.

## References

[1] Union for International Cancer Control (UICC). Tumor Node Metastasis (TNM) Classification of Malignant Tumours. In: Brierley JD Gospodarowicz MK Wittekind C, editors. Oxford, UK; Hoboken, NJ, Wiley-Blackwell (2016).

[2] Lodder WL Lange CAH Teertstra HJ Pameijer FA Van Den Brekel MWM Balm AJM. Value of MR and CT Imaging for Assessment of Internal Carotid Artery Encasement in Head and Neck Squamous Cell Carcinoma. Int J Surg Oncol (2013) 2013:968758. doi: 10.1155/2013/968758 23431430 PMC3569906

[3] Freeman SB Hamaker RC Borrowdale RB Huntley TC. Management of Neck Metastasis With Carotid Artery Involvement. Laryngoscope (2004) 114:20–4. doi: 10.1097/00005537-200401000-00003 14709989

[4] Németh Z Dömötör G Tálos M Barabás J Ujpál M Szabó G. Resection and Replacement of the Carotid Artery in Metastatic Head and Neck Cancer: Literature Review and Case Report. Int J Oral Maxillofac Surg (2003) 32:645–50. doi: 10.1054/ijom.2002.0419 14636618

[5] Roh JL Ra Kim M Choi SH Hyun Lee J Cho KJ Yuhl Nam S. Can Patients With Head and Neck Cancers Invading Carotid Artery Gain Survival Benefit From Surgery? Acta Otolaryngol (2008) 128:1370–4. doi: 10.1080/00016480801968518 18607928

[6] Yousem DM Hatabu H Hurst RW Seigerman HM Montone KT Weinstein GS. Carotid Artery Invasion by Head and Neck Masses: Prediction With MR Imaging. Radiology (1995) 195:715–20. doi: 10.1148/radiology.195.3.7754000 7754000

[7] Yoo GH Hocwald E Korkmaz H Du W Logani S Kelly JK. Assessment of Carotid Artery Invasion in Patients With Head and Neck Cancer. Laryngoscope (2000) 110:386–90. doi: 10.1097/00005537-200003000-00010 10718424

[8] Pons Y Ukkola-Pons E Clément P Gauthier J Conessa C. Relevance of 5 Different Imaging Signs in the Evaluation of Carotid Artery Invasion by Cervical Lymphadenopathy in Head and Neck Squamous Cell Carcinoma. Oral Surgery Oral Med Oral Pathol Oral Radiol Endodontol (2010) 109:775–8. doi: 10.1016/j.tripleo.2009.12.053 20416537

[9] Zaragoza L Sendra F Solano J Garrido V Martínez-Morillo M. Ultrasonography is More Effective Than Computed Tomography in Excluding Invasion of the Carotid Wall by Cervical Lymphadenopathies. Eur J Radiol (1993) 17:191–4. doi: 10.1016/0720-048X(93)90102-S 8293747

[10] Rapoport A Tornin ODS Beserra IM De Neto PBC De Souza RP. Assessment of Carotid Artery Invasion by Lymph Node Metastasis From Squamous Cell Carcinoma of Aero-Digestive Tract. Braz J Otorhinolaryngol (2008) 74:79–84. doi: 10.1016/S1808-8694(15)30755-2 18392506 PMC9450667

[11] Suárez C Fernández-Alvarez V Hamoir M Mendenhall WM Strojan P Quer M. Carotid Blowout Syndrome: Modern Trends in Management. Cancer Manag Res (2018) 10:5617–28. doi: 10.2147/CMAR.S180164 PMC6239123 30519108

[12] Swain RE Biller HF Ogura JH Harvey JE. An Experimental Analysis of Causative Factors and Protective Methods in Carotid Artery Rupture. Arch Otolaryngol (1974) 99:235–41. doi: 10.1001/archotol.1974.00780030245001 4131800

[13] Feiz-Erfan I Han PP Spetzler RF Lanzino G Ferreira MAT Gonzalez LF. Salvage of Advanced Squamous Cell Carcinomas of the Head and Neck: Internal Carotid Artery Sacrifice and Extracranial-Intracranial Revascularization. Neurosurg Focus (2003) 14:1–5. doi: 10.3171/foc.2003.14.3.7 15709723

[14] Kalani MYS Kalb S Martirosyan NL Lettieri SC Spetzler RF Porter RW. Cerebral Revascularization and Carotid Artery Resection at the Skull Base for Treatment of Advanced Head and Neck Malignancies. J Neurosurg (2013) 118:637–42. doi: 10.3171/2012.9.JNS12332 23082880

[15] Nayak UK Donald PJ Stevens D. Internal Carotid Artery Resection for Invasion of Malignant Tumors. Arch Otolaryngol Head Neck Surg (1995) 121:1029–33. doi: 10.1001/archotol.1995.01890090067013 7646855

[16] Couldwell WT Macdonald JD Taussky P. Complete Resection of the Cavernous Sinus - Indications and Technique. World Neurosurg (2014) 82:1264–70. doi: 10.1016/j.wneu.2013.08.026 23994071

[17] Aslan I Hafiz G Baserer N Yazicioglu E Kiyak E Tinaz M. Management of Carotid Artery Invasion in Advanced Malignancies of Head and Neck: Comparison of Techniques. Ann Otol Rhinol Laryngol (2002) 111:772–7. doi: 10.1177/000348940211100902 12296329

[18] Muhm M Grasl MC Burian M Exadaktylos A Staudacher M Polterauer P. Carotid Resection and Reconstruction for Locally Advanced Head and Neck Tumors. Acta Otolaryngol (2002) 122:561–4. doi: 10.1080/00016480260092417 12206270

[19] Katsuno S Takemae T Ishiyama T Usami SI. Is Carotid Reconstruction for Advanced Cancer in the Neck a Safe Procedure? Otolaryngol Head Neck Surg (2001) 124:222–4. doi: 10.1067/mhn.2001.112482 11226961

[20] Rennert RC Ravina K Strickland BA Bakhsheshian J Fredrickson VL Russin JJ. Complete Cavernous Sinus Resection: An Analysis of Complications. World Neurosurg (2018) 119:89–96. doi: 10.1016/j.wneu.2018.07.206 30075273

[21] Jacobs JR Korkmaz H Marks SC Kline R Berguer R. One Stage Carotid Artery Resection: Reconstruction in Radiated Head and Neck Carcinoma. Am J Otolaryngol Head Neck Med Surg (2001) 22:167–71. doi: 10.1053/ajot.2001.23449 11351284

[22] Sekhar LN Natarajan SK Ellenbogen RG Ghodke B. Cerebral Revascularization for Ischemia, Aneurysms, and Cranial Base Tumors. Neurosurgery (2008) 62:1373–410. doi: 10.1227/01.NEU.0000315873.41953.74 18695558

[23] Dare AO Gibbons KJ Gillihan MD Guterman LR Loree TR Hicks WL. Hypotensive Endovascular Test Occlusion of the Carotid Artery in Head and Neck Cancer. Neurosurg Focus (2003) 14:1–4. doi: 10.3171/foc.2003.14.3.6 15709722

[24] Standard SC Ahuja A Guterman LR Chavis TD Gibbons KJ Barth AP. Balloon Test Occlusion of the Internal Carotid Artery With Hypotensive Challenge. Am J Neuroradiol (1995) 16:1453–8. PMC8338089 7484632

[25] Yokoyama J Yazawa M Yoshimoto H Matsuo S Ohba S. Advantages of Superficial Femoral Vein Grafts for Carotid Artery Reconstruction Following Carotid Artery Resection in the Treatment of Head and Neck Cancer. Acta Otolaryngol (2015) 135:302–6. doi: 10.3109/00016489.2014.956336 25649887

[26] Ghi MG Paccagnella A Ferrari D Foa P Alterio D Codecá C. Induction TPF Followed by Concomitant Treatment Versus Concomitant Treatment Alone in Locally Advanced Head and Neck Cancer. A Phase II-III Trial. Ann Oncol (2017) 28:2206–12. doi: 10.1093/annonc/mdx299 28911070

[27] Izawa N Onozawa Y Hikosaka T Hamauchi S Tsushima T Todaka A. Efficacy and Feasibility of Docetaxel, Cisplatin, and 5-Fluorouracil Induction Chemotherapy for Locally Advanced Head and Neck Squamous Cell Carcinoma Classified as Clinical Nodal Stage N2c, N3, or N2b With Supraclavicular Lymph Node Metastases. Int J Clin Oncol (2015) 20:455–62. doi: 10.1007/s10147-014-0749-4 25248339

[28] Haddad RI Posner M Hitt R Cohen EEW Schulten J Lefebvre JL. Induction Chemotherapy in Locally Advanced Squamous Cell Carcinoma of the Head and Neck: Role, Controversy, and Future Directions. Ann Oncol (2018) 29:1130–40. doi: 10.1093/annonc/mdy102 PMC5961254 29635316

[29] Sher DJ Posner MR Tishler RB Sarlis NJ Haddad RI Holupka EJ. Relationship Between Radiation Treatment Time and Overall Survival After Induction Chemotherapy for Locally Advanced Head-and-Neck Carcinoma: A Subset Analysis of TAX 324. Int J Radiat Oncol Biol Phys (2011) 81:e813–8. doi: 10.1016/j.ijrobp.2010.12.005 21300455

[30] Manzoor NF Russell JO Bricker A Koyfman S Scharpf J Burkey B. Impact of Surgical Resection on Survival in Patients With Advanced Head and Neck Cancer Involving the Carotid Artery. JAMA Otolaryngol Head Neck Surg (2013) 139:1219–25. doi: 10.1001/jamaoto.2013.4917 24077023

[31] Dorth JA Patel PR Broadwater G Brizel DM. Incidence and Risk Factors of Significant Carotid Artery Stenosis in Asymptomatic Survivors of Head and Neck Cancer After Radiotherapy. Head Neck (2014) 36:215–9. doi: 10.1002/hed.23280 PMC4445731 23554082

[32] Jacobi C Gahleitner C Bier H Knopf A. Chemoradiation and Local Recurrence of Head and Neck Squamous Cell Carcinoma and the Risk of Carotid Artery Blowout. Head Neck (2019) 41:3073–9. doi: 10.1002/hed.25796 31070287

[33] Dionisi F Fiorica F D’Angelo E Maddalo M Giacomelli I Tornari E. Organs at Risk’s Tolerance and Dose Limits for Head and Neck Cancer Re-Irradiation: A Literature Review. Oral Oncol (2019) 98:35–47. doi: 10.1016/j.oraloncology.2019.08.017 31536844

[34] Garg S Kilburn JM Lucas JT Randolph D Urbanic JJ Hinson WH. Reirradiation for Second Primary or Recurrent Cancers of the Head and Neck: Dosimetric and Outcome Analysis. Head Neck (2016) 38:E961–9. doi: 10.1002/hed.24136 PMC9176709 25993910

[35] Tinganelli W Durante M. Carbon Ion Radiobiology. Cancers 2020 Vol 12 Page 3022 (2020) 12:3022. doi: 10.3390/CANCERS12103022 PMC7603235 33080914

[36] Sulaiman N Demizu Y Koto M Saitoh J Suefuji H Tsuji H. Multicenter Study of Carbon-Ion Radiation Therapy for Adenoid Cystic Carcinoma of the Head and Neck: Subanalysis of the Japan Carbon-Ion Radiation Oncology Study Group (J-CROS) Study (1402 Hn). Int J Radiat Oncol Biol Phys (2018) 100:639–46. doi: 10.1016/J.IJROBP.2017.11.010 29413278

[37] Morimoto K Demizu Y Hashimoto N Mima M Terashima K Fujii O. Nibu K. Particle Radiotherapy Using Protons or Carbon Ions for Unresectable Locally Advanced Head and Neck Cancers With Skull Base Invasion. Jpn J Clin Oncol (2014) 44:428–34. doi: 10.1093/JJCO/HYU010 24620027

[38] Ronchi S Vischioni B Bonora M Barcellini A Locati LD Castelnuovo P. Managing Locally Advanced Adenoid Cystic Carcinoma of the Head and Neck During the COVID-19 Pandemic Crisis: Is This the Right Time for Particle Therapy? Oral Oncol (2020) 106:104803. doi: 10.1016/J.ORALONCOLOGY.2020.104803 32410826 PMC7221390

[39] Jensen AD Poulakis M Nikoghosyan AV Chaudhri N Uhl M Münter MW. Re-Irradiation of Adenoid Cystic Carcinoma: Analysis and Evaluation of Outcome in 52 Consecutive Patients Treated With Raster-Scanned Carbon Ion Therapy. Radiother Oncol (2015) 114:182–8. doi: 10.1016/j.radonc.2015.01.002 25640299

[40] Held T Windisch P Akbaba S Lang K El Shafie R Bernhardt D. Carbon Ion Reirradiation for Recurrent Head and Neck Cancer: A Single-Institutional Experience. Int J Radiat Oncol Biol Phys (2019) 105:803–11. doi: 10.1016/j.ijrobp.2019.07.021 31349059

